# High Occurrence of Multidrug-Resistant *Escherichia coli* Strains in Bovine Fecal Samples from Healthy Cows Serves as Rich Reservoir for AMR Transmission

**DOI:** 10.3390/antibiotics12010037

**Published:** 2022-12-26

**Authors:** Amna Jalil, Shabana Gul, Muhammad Faraz Bhatti, Muhammad Faisal Siddiqui, Fazal Adnan

**Affiliations:** 1 Atta ur Rahman School of Applied Biosciences (ASAB), National University of Sciences and Technology (NUST), Islamabad 44000, Pakistan; 2 Department of Microbiology, Hazara University, Mansehra 21300, Pakistan

**Keywords:** fecal *E. coli* strains, bovine, virulence-associated genes, AMR, β-lactamase

## Abstract

Objectives: Antibiotics are valuable therapeutics. However, the unwarranted and excessive use of these antimicrobials in food animals and the consequent contamination of the environment have been associated with the emergence and spread of antimicrobial resistance. Continuous surveillance and monitoring of antimicrobial resistance among *E. coli* isolates is recommended, not only for bovine health but also for public health. This study aims to assess the antimicrobial resistance profile, virulence potential, and genetic characterization of fecal *E. coli* isolates from healthy cows. Methodology: The in vitro, phenotypic antibiotic resistance of isolates was measured via the Kirby–Bauer disc-diffusion method against twenty-seven antibiotics. The β-lactamase enzymatic activities of the strains were also investigated. For the assessment of virulence potential, fecal *E. coli* isolates were subjected to several in vitro pathogenicity assays, including biofilm formation ability, blood hemolysis, complement resistance, and growth in human urine. Phylogroup determination and virulence-associated genes were detected via multiplex PCR. Results: In vitro antibiotic resistance profiling showed that 186/200 (93%) of the isolates were multidrug-resistant (MDR), with the highest resistance against penicillin, tetracycline, fluoroquinolone, and macrolide classes of antibiotics. Of particular concern was the phenotypic resistance to colistin in 52/200 isolates (26%), though 16% of the total isolates harbored *mcr1*, the genetic determinant of colistin. Despite the scarce use of fluoroquinolone, cephalosporin, and carbapenem in the agricultural sector, resistance to these classes was evident due to the presence of extended-spectrum β-lactamase (ESBL) in 41% of *E. coli* isolates. The β-lactamase genotyping of *E. coli* isolates showed that 47% of isolates harbored either *bla*CTX or *bla*TEM. Approximately 32% of isolates were resistant to serum complement, and their growth in human urine was evident in 18% of isolates, indicating a possible infection of these isolates in high nitrogenous condition. Phylogrouping showed that the most prevalent phylogenetic group among fecal *E. coli* isolates was phylogroup B1 (57%), followed by phylogroups A (33%), D (6%), and B2 (4%). The most prevalent virulence-associated genes in fecal *E. coli* were *fimH*, *iss* and *tatT*. Results showed that ten isolates (5%) harbored the *stx1* gene, the genetic marker of enterohemorrhagic *E. coli.* This study provides insights into the antibiotic resistance and virulence profiling of the fecal *E. coli* isolates from healthy cows. These results emphasize the need for imposing regulations on the proper use of antibiotics and growth promoters in food-producing animals.

## 1. Introduction

Antibiotics are indispensable for the health management and life support of humans and animals, and have been used for the treatment of various infectious bacterial diseases. However, antibiotic-resistant bacteria can arise from unwarranted and excessive use of these antibiotics in animals and humans and can be discharged into the environment in the form of hazardous microbes through feces [1,2,3,4,5]. Currently, antibiotic-resistant bacteria (ARBs) are a major threat around the globe. It is estimated that the annual global death toll due to ARBs will increase up to 10 million by the year 2050 [6]. According to the National Center for Global Health and Medicine (NCGM), 8000 deaths occur annually in Japan due to fluoroquinolone-resistant *Escherichia coli* and methicillin-resistant *Staphylococcus aureus* [7].

In poultry and livestock production, antibiotic usage is very common for the treatment of infections, in sub-therapeutic levels in animal feed, as growth-promoting agents, for disease prevention, and for the improvement of feed-conversion efficiency [8,9,10]. In fact, the amount of antibiotics used in the agricultural setup is much higher than in the clinical setup. Even developed meat-producing countries such as the U.S.A, Brazil, and China use huge amounts of antibiotics during meat production, and the situation is even worse in developing countries where there is no government-level restriction on the use of antibiotics [11]. The consequence of overuse and the incomplete dosing of antibiotics for longer periods puts a selection pressure on bacteria, allowing them to procure antibiotic-resistant genes [12]. Of greater concern is that the antibiotics used in livestock farms are mostly the counterparts of those used for humans (meaning that they may belong to the same class and act in a similar manner), leading to the transmission of the resistant genes when human–animal interface occurs [11,13,14].

Thus, livestock farms are considered to be potential reservoirs of ARBs that can be transferred to humans via food consumption (milk and meat) [15], through direct or indirect contact with the infected animals, or through contact with the excretory material of animals such as urine, feces, or blood. It has been observed that a large number of antibiotics are not transformed into inactive forms when given to animals and are instead deposited into the animals’ tissues or excreted out into the environment, creating another reservoir of ARBs [16]. Some studies have confirmed that antibiotic-resistant *E. coli* and other ARBs can spread to the natural rivers and other water bodies from livestock wastewater [17] and can also be transported to other natural environments via rodents and other small animals [18,19,20]. Several studies confirmed the transmission of ARBs from livestock-derived compost to vegetables and other crops [21,22,23].

The One Health project was an initiative aimed at controlling the issue of antibacterial resistance travelling from animal farms to other environments, clinics, and hospitals. This initiative was a three-pronged system that consisted of humans, animals, and the environment in which they live. However, the environment is the least understood aspect of this system [12]. The environment sector is important, as environmental microbes can serve as a reservoir for the genes responsible for antibiotic resistance [13]. The current study targets this neglected sector of the environment from the One Health triad. As it is ubiquitous in nature as a commensal as well as a pathogen, *E. coli* is one of the major vehicles that can transmit resistance and virulence genes between different species. The current study focuses on the characterization and antibiotic-susceptibility pattern of *E. coli* strains isolated from the fecal samples of healthy cows.

## 2. Methodology

### 2.1. Ethical Statement

All the protocols and experiments of this research were in accordance with the regulations and guidelines of veterinary research ethics. All protocols, including sampling, handling of fecal samples, storage of samples, and analysis of the results, were approved by the Board of Advanced Studies and Research (BASR) of the National University of Sciences and Technology (NUST), Islamabad. In addition, written consent was obtained from the farm handlers before sampling. It was ensured that only fecal sampling was performed, and no experimental work was conducted on the farm animals.

### 2.2. Examination of Cows before Sampling

Prior to fecal sampling, a clinical examination of milk and cow udders was conducted. For the milk examination, milk from each quarter was separately examined by visual inspection for the presence of any blood clots, flakes, coagulates of milk, smell, and color change. For the clinical examination of the udder, the udder of each cow was first examined visually and then by palpation to detect any inflammatory swelling, atrophy of udder tissue, or fibrosis. The consistency and size of the udder were also inspected for any abnormalities such as firmness, disproportional symmetry, and blindness. Furthermore, a California mastitis test was also conducted on the milk to check for subclinical mastitis. Input from the farmers about the health condition of the cows was also considered, and only healthy cows were included in the study.

### 2.3. Isolation of E. coli from Feces of Healthy Cows

Fecal matter from healthy cows was collected from the Rawalpindi district and capital territory. For the collection of fecal samples (200 samples), a swab was dipped in the fresh fecal matter of healthy cows and immersed in peptone water. The mixture was then transported to the laboratory in ice box. For enrichment purposes, 1 mL of mixture was mixed with 9 mL of buffered Peptone water (Oxoid^TM^, Basingstoke, UK) and incubated overnight at 37 °C in a shaking incubator. One loopful of the incubated sample was streaked onto MacConkey agar (Oxoid^TM^) and incubated at 37 °C for 24 h. One lactose-fermenting colony was chosen from the plate and streaked onto EMB agar (Oxoid^TM^). One colony with a characteristic green, metallic sheen was used for further experimentation.

### 2.4. Molecular Identification of E. coli

The DNA of the *E. coli* isolates was extracted by the boiling method. For that purpose, a single, purified colony of the presumptive positive *E. coli* culture was emulsified in 50 µL of molecular grade water for 10 min, followed by centrifugation (10,000× *g* for 10 min). The supernatant was stored at −20 °C, and 2 µL of the supernatant was used as template DNA for PCR. The isolates were confirmed as *E. coli* by PCR detection of the *E. coli*-specific housekeeping gene *uidA* that encodes for enzyme β-glucuronidase and presents in almost 98% of *E. coli* strains [24]. The gene-amplification conditions were as follows: an initial denaturation (95 °C for 5 min), 35 cycles of denaturation (95 °C for 30 s), annealing (55 °C for 30 s), the cyclic extension (72 °C for 1 min), and the final extension step (72 °C for 10 min).

### 2.5. Antimicrobial Susceptibility Testing

In accordance with the recommendations of the Clinical Laboratory Standards Institute [25], the Kirby–Bauer disc-diffusion method was used to investigate the antibiotic-susceptibility patterns of the bovine fecal *E. coli* isolates. A total of 26 antibiotics were procured from Oxoid™. The diameters of the zones of inhibition were measured in millimeters and were categorized as susceptible (S), intermediate (I), or resistant (R). The antibiotics, along with their measurements of zones of inhibition are mentioned in Supplementary Table S1. *E. coli* ATCC-25922 was used as an antibiotic-sensitive control.

### 2.6. Determination of Minimum Inhibitory Concentration (MIC) of Colistin

*E. coli* isolates were inoculated in Mueller–Hinton broth (MHB) (Oxoid^TM^) in a range of colistin concentrations ranging from 0.125 µg/mL to 128 µg/mL. The lowest colistin concentration that entirely prevented *E. coli* from visible growth was identified as the MIC endpoint. Colistin-sensitive and colistin-resistant *E. coli* isolates were distinguished by an MIC of ≤4 μg/mL and an MIC of >4 μg/mL, respectively [26,27].

### 2.7. Screening and Confirmation of ESBL Production

*E. coli* isolates that displayed a diameter of ≤27 mm for cefotaxime and/or ≤22 mm for ceftazidime underwent a double-disc synergy Test (DDST) for the confirmation of ESBL production. Any distortion in the zone of inhibition towards AMC or an increase or decrease of 5 mm in the zone of inhibition was regarded as a favorable finding for ESBL production [25].

### 2.8. Screening and Confirmation of Metallo β-Lactamase (MBL) Production

*E. coli* isolates that exhibited resistance to meropenem (MEM, 10 μg), imipenem (IPM, 10 μg), or ceftazidime (CAZ, 30 μg) were screened out for the confirmation of MBL production by an inhibition method, which employed EDTA as an inhibitor. An increase of 7 mm in the diameter of the zone of inhibition of any of the combined discs (antibiotic disc supplemented with EDTA) relative to the single one (disc containing only antibiotics) was considered to be a confirmed strain for MBL production [28].

### 2.9. Modified Hodge Test for the Detection of Carbapenemase Production

To detect carbapenemase production by *E. coli* isolates, a modified Hodge test was used. A 0.5 McFarland dilution of the *E. coli* ATCC 25922 was prepared, and a 1:10 dilution of this solution was spread on Mueller–Hinton agar (MHA) (Oxoid™) plates. A 10 µg meropenem disc was positioned in the middle of the plate’s center and the test isolate was streaked in a straight line from close to the disc’s edge to the plate’s edge. Following incubation, a clover-leaf-like indentation of the *E. coli* ATCC 25922 growing along with the test APEC isolates’ growth within the disk-diffusion zone was considered a positive result for carbapenemase production [29].

### 2.10. AmpC Disc Test for the Detection of AmpC β-Lactamase Production

Fecal *E. coli* isolates that yielded a zone diameter <18 mm for Cefoxitin (FOX, 30 μg) were subjected to the AmpC disc test for the confirmation of the AmpC enzyme. On an MHA plate, a cefoxitin disc was placed on top of a lawn of cefoxitin-susceptible *E. coli* ATCC 25922. The AmpC discs were rehydrated with 20 µL saline and inoculated with several colonies of a tested fecal *E. coli* isolate. The resultant inoculated disc was then placed near the FOX disc. The plate was incubated at 37 °C for 24 h and checked for any flattening or indentation of the inhibitory zone, which would indicate the enzymatic inactivation of FOX (positive result) [30].

### 2.11. Determination of Multiple Antibiotic Resistance Index (MARI) and Resistance Score (R-Score)

To establish the multiple-antibiotic-resistance profile of the *E. coli* isolates, MARI was calculated [31]. The formula of MARI was described as:

(1) MARI=a/b

where a represents the number of antibiotics that the tested *E. coli* isolate was resistant to and b represents the total number of antibiotics that the tested *E. coli* isolate was assessed against.

For a given *E. coli* isolate, the R-score was described as the number of antibiotics against which the isolate exhibited intermediate or complete resistance. Resistance scores of 0.5 and 1 were attributed to isolates exhibiting intermediate or complete resistance, respectively, against a given antibiotic [32].

### 2.12. Detection of Antibiotic Resistance Genes

Fecal *E. coli* isolates were scrutinized for colistin-resistance genes through the detection of *mcr-1* and *mcr-2* genes [33]. Clinical isolates *E. coli* CB51 and *E. coli* CB53 were used as positive controls for the *mcr-1* and *mcr-2* genes, respectively. These strains were provided by the Antibacter lab of NUST. The *E. coli* isolates were further examined for ESBL genes (*bla*TEM, *bla*CTX, and *bla*SHV), MBL genes (*bla*NDM, *bla*VIM, and *bla*IMP), and carbapenemase genes (*bla*KPC and *bla*OXA-48) by PCR using primers and conditions described by Doyle [34] (Supplementary Table S1). *Klebsiella pneumoniae* ATCC 700603 was used as a positive control, and *E. coli* ATCC 25922 was used as a negative control for ESBL-gene identification.

### 2.13. In Vitro Pathogenicity Analysis

All bovine, fecal *E. coli* isolates were subjected to phenotypic analysis for biofilm formation ability, Congo red (CR) binding ability, blood hemolysis, lipase activity, protease activity, growth in human urine, complement resistance, and motility assays (swimming, swarming, and twitching).

A biofilm assay for *E. coli* isolates was performed according to the microtiter plate method [35]. The optical density (OD) of isolates was adjusted at 1.0 and diluted 1:100 in tryptone soy broth (TSB). The diluted sample was dispensed in a 96-well plate and incubated at 37 °C for 48 h. Planktonic cells were removed, loosely bound cells were removed by washing with saline, and tightly attached cells were stained with a 0.1% crystal-violet (CV) solution for 10 min. They were then resuspended in 33% glacial acetic acid, and their absorbance was recorded at 595 nm. The biofilm-forming ability of each isolate was calculated with the formula:

ODc: Average OD of negative control + 3 × Standard deviation (SD) of negative control

OD ≤ ODc—no biofilm production

ODc < OD ≤ 2 × ODc—weak biofilm production

2 × ODc < OD ≤ 4 × ODc—moderate biofilm production

4 × ODc < OD—strong biofilm production

*Pseudomonas aeruginosa* and *E. coli* DH5α strains were used as positive and negative controls, respectively.

For the blood hemolysis assay, *E. coli* isolates were streaked on blood agar plates infused with 5% aseptic, fresh, defibrinated sheep blood. The plates were incubated for 24 h at 37 °C.

The Congo Red (CR) binding assay was conducted in accordance with the method described by Berkhoff & Vinal [36]. *E. coli* isolates were streaked on CR agar plates, incubated for 24 h at 37 °C, and then kept at room temperature for another 48 h. Red colonies were identified as CR positive while white, grey, or pink colonies were identified as CR negative.

The bacterial growth in human urine was tested to analyze if the isolates could grow in the high-urea and nitrogenous environment of urine. Some studies suggest that *E. coli* isolates that have the uropathogenic *E. coli* (UPEC) gene and can survive in human urine (*in vitro*) might have the potential to cause urinary tract infections (UTI) in humans upon contact. The assay was performed using the method described by Mitchell et al., 2015 [35] with few modifications. For the collection of urine samples, healthy male and female subjects were asked to sign the form approved by Institutional review board (IRB) committee of the Industrial Biotechnology Department of NUST (Ref No: IRB-88). Urine samples collected from healthy male and female subjects were filter-sterilized (with a 0.2 µm sterile filter), pooled, and stored in aliquots at −20 °C. Briefly, the optical density of the tested isolates was adjusted to 1.0. A and a 1:100 dilution of isolates was prepared in sterile urine. The dilutions were dispensed in a microtiter plate. The plate was incubated for 8 h at 37 °C under static conditions, and the absorbance was recorded at 600 nm. The UPEC strain CFT073 and *E. coli* DH5α strain were used as positive and negative controls, respectively.

The lipase activity of the fecal *E. coli* isolates was checked by streaking the isolates on tryptic soy agar (TSA) supplemented with 1% Tween 80, followed by incubation for 1–2 days at 37 °C. *E. coli* isolates that tested positive for lipase activity were distinguished by clear halo zones around the bacterial growth.

The protease activity of the *E. coli* isolates was determined by streaking the isolates on TSA supplemented with 1% casein from bovine milk (Sigma Aldrich, Schnelldorf, Germany), followed by incubation for 1–2 days at 37 °C. *E. coli* isolates that tested positive for protease activity were identified by clear halo zones around the bacterial growth.

The resistance to serum complement was analyzed by the quantitative microtiter plate method with a slight modification [37]. Briefly, 10^4^ CFU of *E. coli* isolates was mixed with an equal volume of 50% fetal bovine serum and the resultant mixture was dispensed in a 96-well microtiter plate. The plate was incubated at 37 °C for 4 h under static conditions and the OD_492_ was determined. *E. coli* isolates were considered serum-complement-resistant if the OD_492_ in serum-containing wells exceeded or equaled that of the no-serum control well. Heat-inactivated sera were used as a control. UPEC strain CFT073 and *E. coli* K-12 MG1655 strain were used as positive and negative controls, respectively.

Motility assays investigating the swarming, swimming, and twitching patterns of *E. coli* isolates were performed using the methods described earlier [38]. Freshly poured motility media plates were dried at room temperature for 6h and stabbed with tested *E. coli* isolates, followed by incubation at 37 °C. Swimming and swarming motilities were examined by measuring the turbid zones around the bacterial swab. Twitching agar media from the twitching motility plates was removed, and plates were washed and stained with 1% CV for 15 min. Finally, the stained zone was measured for the twitching motility assay. At least six colonies from each *E. coli* isolate were tested to determine their motility pattern. *P. aeuroginosa* was used as a positive control for the motility assay.

### 2.14. Phylogenetic Classification of E. coli Isolates

**Figure 1:**
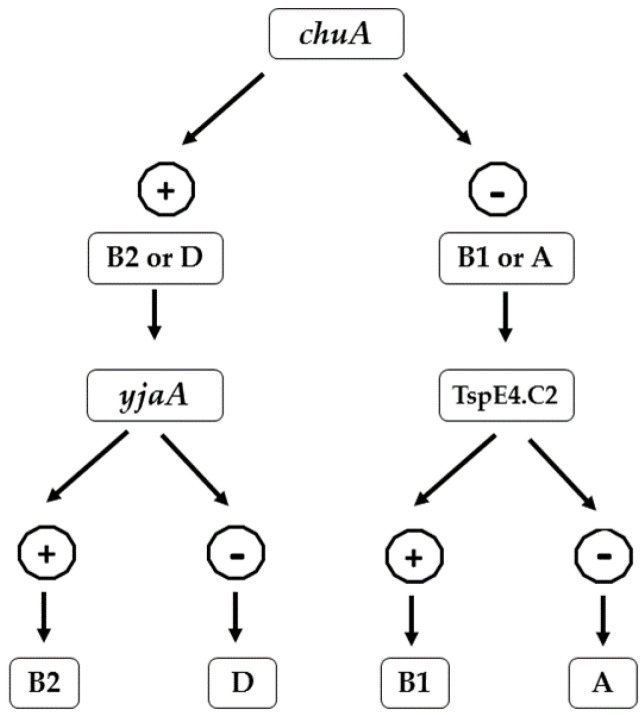
Flowchart showing the scheme of phylogroup determination proposed by Clermont et al. based on the presence and absence of *chuA*, *yjaA,* and DNA fragment TSPE4.C2.

Fecal *E. coli* were classified into different phylogenetic groups (Phylogroups A, B1, B2, and D) using a single, multiplex PCR as described by Clermont [39]. The genomic DNA was used for the amplification of *chuA*, *yjaA*, and DNA fragment TSPE4.C2. Based on the presence or absence of these 3 DNA markers (c*huA*, *yjaAii*, and DNA fragment TSPE4.C2), *E. coli* isolates were assigned to specific phylogenetic groups (Figure 1).

### 2.15. Detection of Virulence-Associated Genes (VAGs)

Several virulence-associated genes were selected for screening in *E. coli* isolates via multiplex PCR using primers and conditions listed in Supplementary Materials. Three replicates were used for each gene identification. UPEC strain CFT073 and some clinical ExPEC strains (provided by Antibacter Lab) were used as positive controls.

### 2.16. Detection of Diarrheagenic E. coli (DEC) Related Genes

To analyze whether fecal *E. coli* has toxin genes or not, *E. coli* isolates were examined for the presence of the DEC virulence-encoding genes mentioned in Table 1. Six groups of DEC were analyzed, including enterohaemorrhagic *E. coli* (EHEC), enteropathogenic *E. coli* (EPEC), enteroinvasive *E. coli* (EIEC), enterotoxigenic *E. coli* (ETEC), and enteroaggregative *E. coli* (EAEC). The primers’ sequencing and their conditions are mentioned in Supplementary Materials. *E. coli* O157:H7 (ATCC 35150) was used as a positive control for the *stx1*, *stx2*, and *eaeA* genes.

## 3. Results

### 3.1. Distribution of Fecal E. coli Isolates

**Figure 2:**
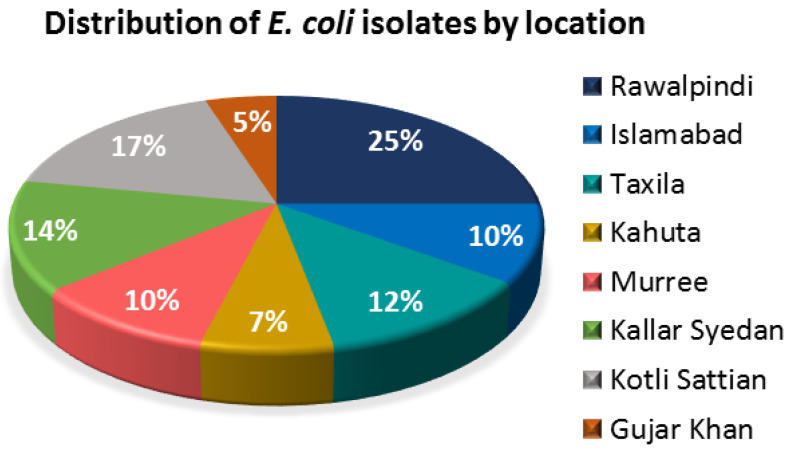
Pie chart showing the distribution of the bovine fecal *E. coli* isolates according to their isolation locations.

A total of 200 isolates from the feces of healthy cows were confirmed to be *E. coli* based on a green, metallic-sheen appearance on EMB agar and a *uidA* gene presence. The relative distribution of these 200 isolates by their isolation locations is summarized in Figure 2, in which most of the *E. coli* strains were isolated from Rawalpindi (25%), followed by Kotli Sattian (17%) and Kahuta (14%). Sampling was carried out from forty different dairy farms of the Rawalpindi district, where five dairy farms were randomly selected from each location for sampling.

### 3.2. In Vitro Antibiotic Susceptibility Pattern of the Bovine Fecal E. coli Isolates

**Figure 3:**
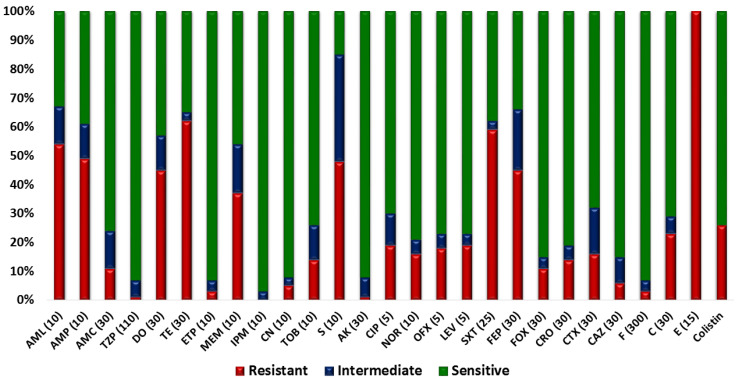
In vitro antibiotic-susceptibility pattern of isolates; a stacked-bar chart showing the percentage resistance and sensitivity of bovine fecal *E. coli* isolates towards tested antibiotics. The antibiotics used in AST profiling include (1) amoxicillin (AML, 10 µg), (2) Ampicillin (AMP, 10 µg), (3) amoxicillin-clavulanate (AMC, 20/10 µg), (4) doxycycline (DO, 30 µg), (5) tetracycline doxycycline (TE, 30 µg), (6) piperacillin-tazobactam (TZP, 100/10 µg), (7) eratapenem (ETP, 10 µg), (8) meropenem (MEM, 10 µg), (9) imipenem (IPM, 10 µg), (10) gentamicin (CN, 10 µg), (11) tobramycin (TOB, 10 µg), (12) streptomycin (S, 10 µg), (13) amikacin (AK, 30 µg), (14) ciprofloxacin (CIP, 5 µg), (15) norfloxacin (NOR, 10 µg), (16) ofloxacin (OFX, 5 µg), (17) levofloxacin (LEV, 5 µg), (18) trimethoprim-sulfamethoxazole (SXT, 25 µg), (19) cefepime (FEP, 30 µg), (20) cefoxitin (FOX, 30 µg), (21) ceftriaxone (CRO, 30 µg), (22) cefotaxime (CTX, 30 µg), (23) ceftazidime (CAZ, 30 µg), (24) nitrofurantoin (F, 300 µg), (25) chloramphenicol (C, 30 µg), (26) erythromycin (E, 15 µg), and (27) colistin sulfate.

**Figure 4:**
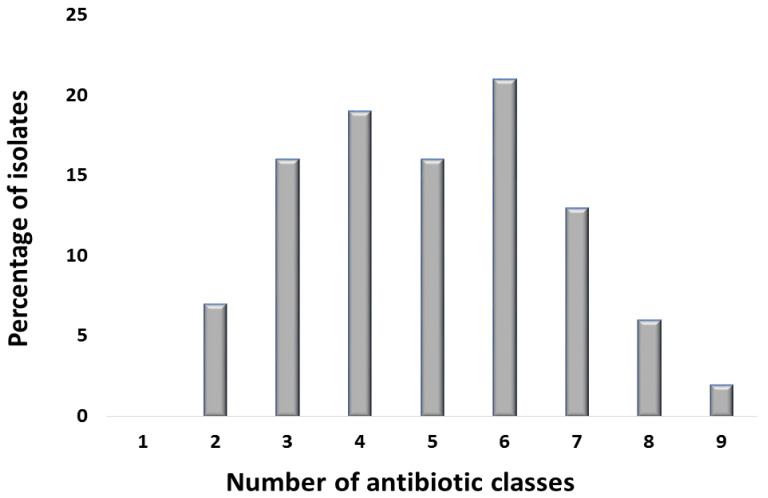
Bar chart showing the number of *E. coli* isolates resistant to different antibiotic classes tested. The x-axis shows the number of antibiotic classes, while y-axis shows the percentage of *E. coli* isolates resistant to specific number of antibiotic classes. The antibiotic classes that were used for antibiotic-susceptibility profiling include: (I) penicillin, (II) tetracycline, (III) penicillin β-lactamase inhibitor, (IV) carbapenem, (V) fluoroquinolone, (VI) aminoglycoside, (VII) cephalosporin, (VIII) nitrofuran, (IX) phenicol, (X) macrolide, (XI) Colistin, and (XII) sulfonamide.

All isolates were resistant to erythromycin that belonged to the macrolide class. The next less-effective class was tetracycline: 124 isolates (62%) were resistant to tetracycline, while 90 isolates (45%) were resistant to doxycycline. Regarding the penicillin class, 108 isolates (54%) were resistant to amoxicillin, while 98 isolates (49%) were resistant to ampicillin. Around 59% of the isolates were resistant to the Trimethoprim/sulfamethoxazole that belonged to the sulphonamide + diaminopyrimidine class. As far as the fluoroquinolone class was concerned, 38 isolates (19%) were resistant to levofloxacin and ciprofloxacin each, 36 isolates (18%) showed resistance towards ofloxacin, and 32 isolates (16%) were resistant to norfloxacin. Regarding second-generation cephalosporin, cefoxitin showed resistance against 22 isolates (11%). Third-generation cephalosporins were less effective as 12 isolates (6%) were resistant to ceftazidime, 28 isolates (14%) were resistant to ceftriaxone, and 32 isolates (16%) were resistant to cefotaxime. Regarding fourth-generation cephalosporin, 90 isolates (45%) showed resistance against cefepime. Resistance to chloramphenicol, representing the phenicol class, was exhibited by 46 isolates (23%), and 52 isolates (26%) were resistant to colistin (Figure 3). Furthermore, 186 isolates (93%) were classified as MDR as they were resistant to more than three antibiotic classes (Figure 4).

### 3.3. Location and Phylogenetic Groups Affecting Variability in MAR Index and R-Score

**Figure 5:**
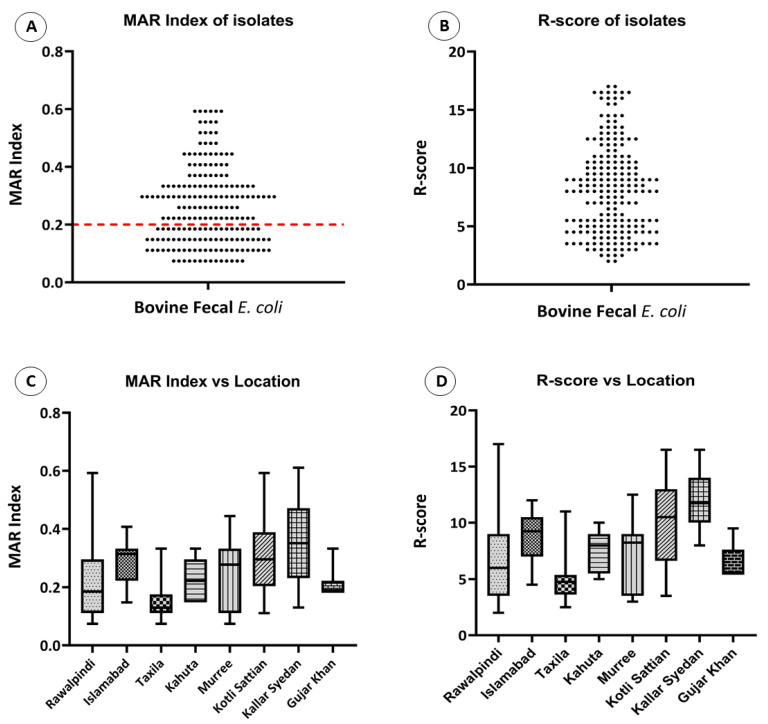
Phenotypic associations. (**A**) Scatter plot showing the MAR index of bovine fecal *E. coli* isolates. Dots representing each isolate’s response show the variability and distribution of data. The isolates above the red line are considered highly resistant, (**B**) scatter plot showing the R-Score of *E. coli* isolates. Dots representing each isolate’s response show the variability and distribution of data, (**C**) box plot showing the comparison of the MAR index of various locations from which the bovine fecal *E. coli* isolates were collected, and (**D**) box plot showing the comparison of R-Scores of various locations from which the *E. coli* isolates were collected.

For further analyses and comparisons, a resistance score (R Score) and MAR index were defined. Overall, 116 *E. coli* isolates (58%) showed a MAR index above 0.2 that is the indicative of high resistance in that specific isolate (Figure 5A,B). To test whether the location of the isolates had any effect on the level of resistance, their resistance score and MAR index were compared. Overall, the highest MAR index was observed in samples from Kallar Syedan, followed by Islamabad and Kotli Sattian. The lowest MAR index was observed in the samples collected from Taxila, followed by Rawalpindi and Gujar Khan (Figure 5C). The R-score showed the same results as the MAR index; the highest R-Score was observed in samples from Kallar Syedan, followed by Kotli Sattian. The lowest R-Score was observed in the samples collected from Taxila (Figure 5D).

**Figure 6:**
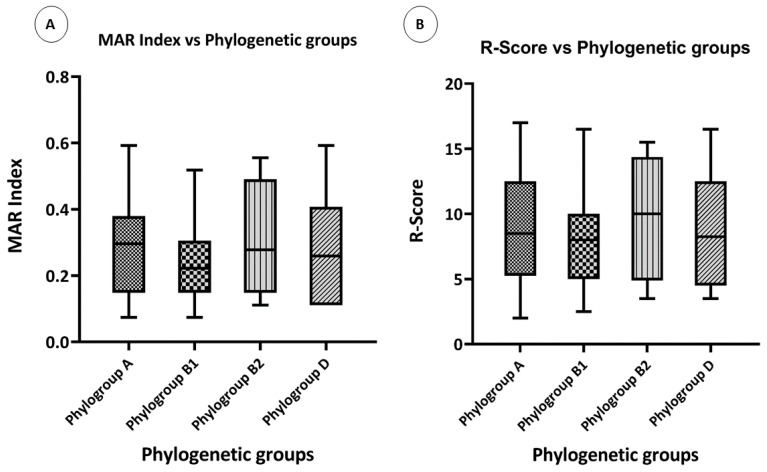
Phenotypic associations: (**A**) box plot showing the comparison of the MAR indexes of various phylogenetic groups, and (**B**) box plot showing the comparison of the R-Scores of various phylogenetic groups.

To test whether the phylogenetic groups of the isolates had any effect on the level of resistance, their resistance scores and MAR indexes were compared. Overall, the highest MAR index was observed in phylogenetic group A, followed by phylogroup B2. The lowest MAR index was observed in phylogroup B1 (Figure 6A). The highest R-score was observed in phylogroup B2, and the rest of the three phylogroups had almost the same R-score (Figure 6B).

### 3.4. ESBL, MBL, AmpC β-Lactamase, and Carbapenamase Activites of the Bovine Fecal E. coli Isolates

When a DDST test was applied to the *E. coli* isolates, 82 isolates (41%) showed positive synergism indicative of ESBL activity (Table 2). Some *E. coli* isolates showed resistance to more than one cephalosporin antibiotic, which proposed that they might be expressing more than one ESBL gene. When the EDTA inhibition test was applied, 16 isolates (8%) showed inhibition in the presence of EDTA (Table 2). The AmpC disc test showed that only 6 isolates (3%) demonstrated AmpC β-lactamase activity (Table 2). Only 2 isolates (1%) showed carbapenemase activity in *E. coli* isolates, as was confirmed by the modified Hodge test.

### 3.5. Detection of Antibiotic Resistance Genes in the E. coli Isolates

Overall, 85 isolates (42.5%) were positive for *bla*TEM presence. while *bla*CTX was detected in 19 isolates (9.5%). A total of 10 isolates (5%) were positive for both *bla*TEM and *bla*CTX genes. All fecal *E. coli* isolates were negative for *bla*NDM, *bla*SHV, *bla*IMP, *bla*VIM, and *bla*KPC. Furthermore, 32 isolates (16%) were positive for the *mcr-1* gene and all isolates were negative for the *mcr-2* gene.

### 3.6. In Vitro Pathogenicity Analysis of E. coli Isolates

**Figure 7:**
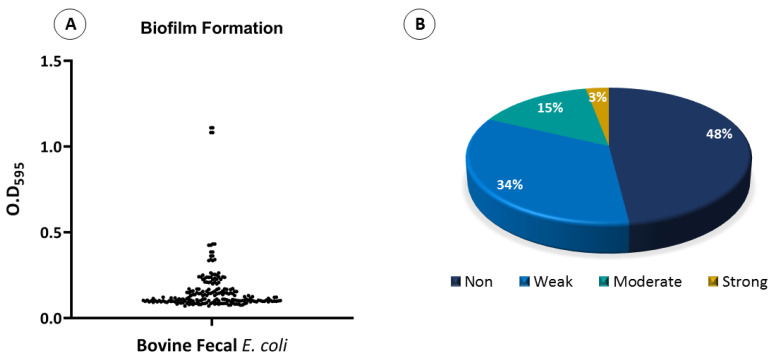
Biofilm formation assay (**A**) scatter plot showing the biofilm formation by bovine fecal *E. coli* isolates. Dots represent each isolate’s biofilm value. (**B**) Pie chart showing the percentage of biofilm formation by bovine fecal *E. coli* isolates.

**Figure 8:**
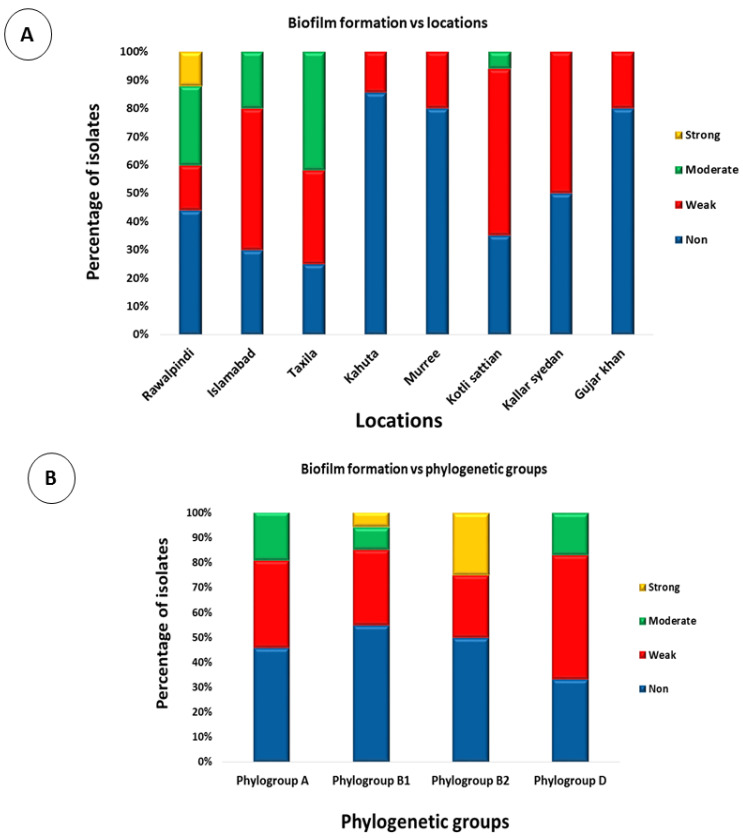
Phenotypic associations: (**A**) stacked bar chart showing the comparison of biofilm formation by *E. coli* strains isolated from various locations, and (**B**) stacked bar chart showing the comparison of biofilm formation by *E. coli* strains from different phylogenetic groups.

The biofilm-formation assay confirmed that 96 *E. coli* isolates (48%) were unable to form a biofilm, 68 *E. coli* isolates (34%) exhibited weak biofilm formation, 30 isolates (15%) formed moderate biofilms, and 6 isolates (3%) exhibited strong biofilm formation (Figure 7). To test whether the location of the isolates had any effect on the level of biofilm formation, their biofilm formation results were compared. Overall, the only strains that showed strong biofilm formations were those that were isolated from Rawalpindi. The least biofilm formation was observed in strains isolated from Kahuta, followed by Murree and Gujar Khan (Figure 8A). To test whether the phylogenetic groups of the isolates had any effect on the level of biofilm formation, their biofilm formation results were compared. Overall, strains from phylogroup B2 showed the highest biofilm formation, and the least biofilm formation was observed in strains from phylogroup A, followed by phylogroup D (Figure 8B).

**Figure 9:**
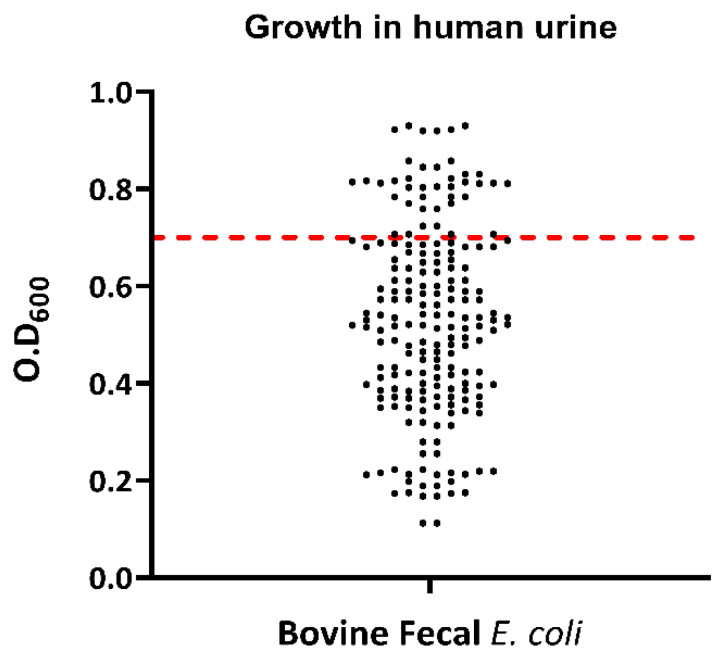
Scatter plot showing the growth in human urine by bovine fecal *E. coli* strains isolated from healthy cows. Dots represent each isolate’s optical-density value and the red line represents the standard O.D value required by the strains to be determined positive for growth in human urine; the strains at or above the red lines are positive for growth in human urine.

Growth in human urine was evident in 36 (18%) of the 200 tested *E. coli* isolates (Figure 9). For the Congo red binding assay, a total of 62 isolates (31%) were able to bind to the Congo red dye, as was indicated by the red appearance of *E. coli* colonies on CRA-streaked plates. A total of 138 isolates (69%) remained unbound to the Congo red dye, as was indicated by the greyish/pinkish growth of those isolates on Congo red media. All *E. coli* isolates showed γ-hemolysis. Overall, 64 (32%) of the 200 tested *E. coli* isolates were complement-resistant as their OD_492_ in serum-containing wells exceeded or equaled that of the no-serum control wells after 4 h of growth. All bovine fecal *E. coli* isolates were negative for lipase and protease activity.

**Figure 10:**
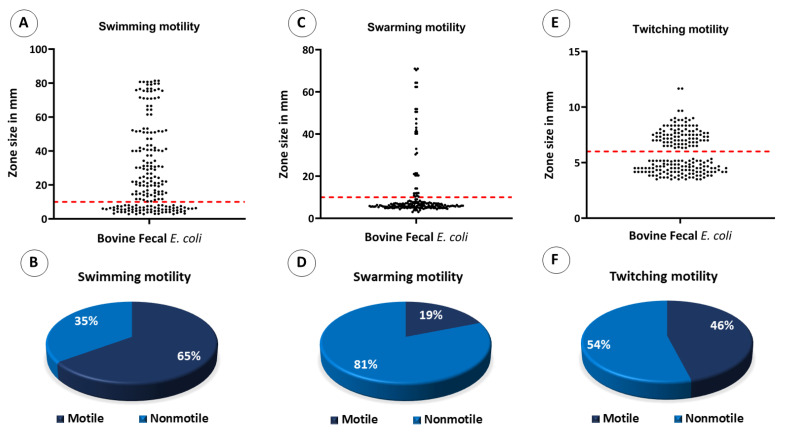
Motility assays by bovine fecal *E. coli* isolates. Dots in scatter plots represent each isolate’s motility in its respective media, and the red line represents the standard cutoff value required by the strains to be determined motile; the strains at or above the red lines are motile. (**A**) Scatter plot showing swimming motility by bovine fecal *E. coli* isolates, (**B**) pie chart showing the percentage of isolates that were positive and negative for swimming motility, (**C**) scatter plot showing swarming motility by bovine fecal *E. coli* isolates, (**D**) pie chart showing the percentage of isolates that were positive and negative for swarming motility, (**E**) scatter plot showing twitching motility bovine fecal *E. coli* isolates, and (**F**) pie chart showing the percentage of isolates that were positive and negative for twitching motility.

As far as swarming and swimming motilities were concerned, those *E. coli* isolates that were considered motile had a turbid zone above 10 mm. Overall, 130 (65%) of the 200 *E. coli* isolates showed swimming motility (Figure 10A,B). For swarming motility, only 38 isolates (19%) out of 200 tested *E. coli* isolates were motile (Figure 10C,D). For twitching motility, those isolates that were considered motile had a stained zone size of ≥ 6 mm. Overall, 92 (46%) of the 200 *E. coli* isolates exhibited twitching motility (Figure 10E,F).

### 3.7. Phylogenetic Classification of E. coli Isolates

**Figure 11:**
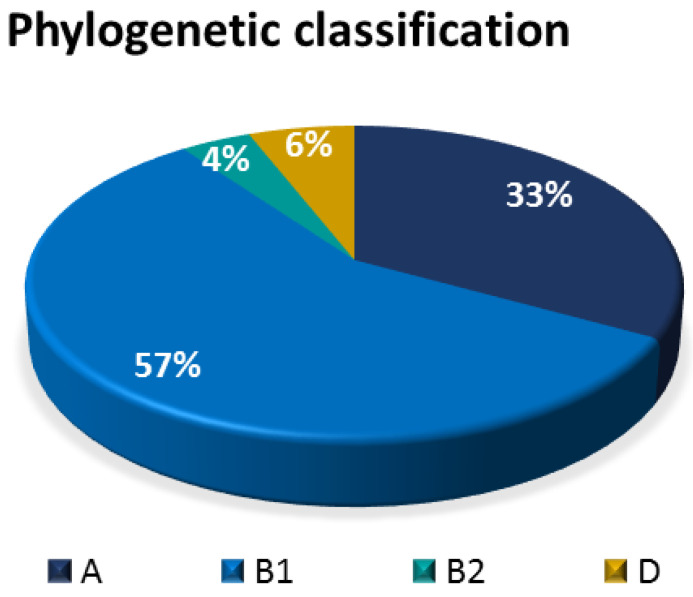
Pie chart showing the relative distribution of phylogenetic groups among bovine fecal *E. coli* isolates.

The *E. coli* isolates were phylogenetically classified into four groups, namely: A, B1, B2, and D. Results showed that the most prevalent phylogenetic group was B1 (57%),s followed by A (33%), D (6%), and B2 (4%) (Figure 11).

### 3.8. Prevalence of VAGs

**Figure 12:**
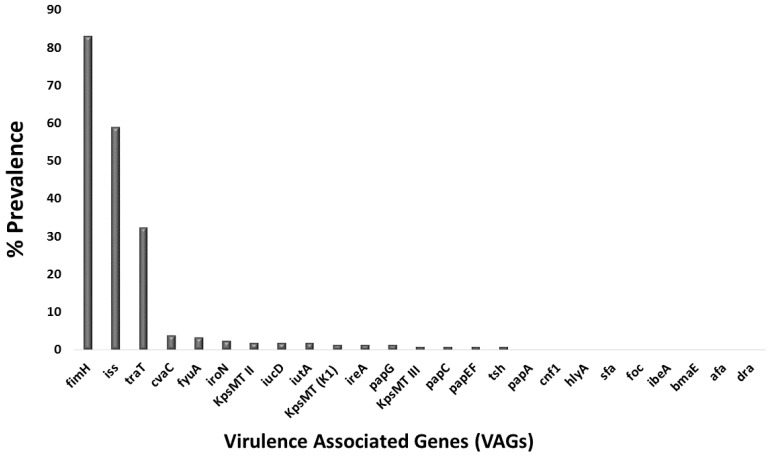
Bar graph showing the percentage prevalence of virulence-associated genes in bovine fecal *E. coli* isolates.

Virulence genotyping of two hundred fecal *E. coli* isolates identified sixteen of the twenty-five studied VAGs in at least one isolate each. The nine exceptions were the genes for certain adhesins (*papA*, *ibeA*, *foc, sfa*, *bmaE*, *afa*, and *dra*) and toxins (*hlyA* and *cnf1*). Among the sixteen detected genes, prevalence values ranged from 1% to 83%. At a very low prevalence (1 to 10%) were certain adhesins: *papC* (1%), *papEF* (1%), *papG* (1.5%), & *cvaC* (4%), Protectins: *KpsMT II* (2%), *KpsMT (K1)* (1.5%), & *KpsMT III* (1%), autotransporter *tsh* (1%), siderophore *ireA* (1.5%), *iucD* (2%), *iutA* (2%), *iroN* (2.5%), & *fyuA* (3.5%). None of the VAGs were found at a low prevalence (11 to 30%). At a medium prevalence (31 to 60%) were protectins *traT* (32.5%) and *iss* (59%). Finally, at the highest prevalence (>60%) was the adhesin *fimH* (83%) (Figure 12).

### 3.9. Frequency of DEC Markers

Among the 200 fecal *E. coli* isolates evaluated, 10/200 isolates harbored the *stx1* gene. No other DEC marker was detected in tested isolates.

## 4. Discussion

*E. coli* naturally reside in mammalian intestines in a commensal form, but they have a tendency to acquire certain VAGs and can cause a variety of intestinal and extra-intestinal infections that result in high morbidity and mortality around the globe [40]. It is speculated that *E. coli* will be transformed into incurable bacterial strain by 2050, as the bacteria are likely to develop resistance against many antibiotic classes by that time [6]. In developing countries such as Pakistan, antibiotics are extensively and irrationally used in food-producing-animal farms for infection control and as growth promoters. This continuous exposure employs selection on the bacteria and is also enhanced by the bacterial ability to attain other resistance factors from the surrounding bacteria, leading to the appearance of modified strains with diverse resistance traits [41].

Despite these concerns, the global consumption of antibiotics by food animals is expected to increase by 67% between 2010–2030 [42]. This study aimed to determine the antimicrobial-resistance profiles, virulence potential, and frequency of virulence related genes in bovine fecal *E. coli* strains isolated from healthy cows from several regions of Pakistan.

The resistance pattern of strains is widely dependent on the geographical location or the animal from which they are isolated. In this study, the highest resistance was found against penicillin, tetracycline, macrolide, and fluoroquinolone, suggesting the overuse of these antibiotic classes in the dairy farms as growth promoters and to treat. Several other studies with *E. coli* from bovine sources also showed antimicrobial resistance to these antibiotics [43,44,45,46,47,48]. The combined resistance of these antibiotics may be due to the co-location of various determinants in the same mobile genetic elements (transposons, plasmids, and/or integrons), and this has contributed to the selection of multidrug-resistant isolates globally [45]. In this study, the prevalence of MDRs (93%) among the tested isolates was higher than previous studies worldwide, for example: 8% in the manure pits of dairy farms in Canada [49], 13.7% in South Africa [50], 37.1% in Jordan [46], 44.4% in Egypt [51], 56.0% in France [52], and 69.0% in Portugal [53]. Some recent studies showed a higher prevalence of MDR *E. coli,* including a 95.6% prevalence in the poultry farms in China [54] and a 100% prevalence of MDR *E. coli* in various environmental samples from Pakistan [55]. This might be due to inappropriate or excessive use of antibiotics for prophylactic and therapeutic purposes in this region.

Another great concern is the presence of the mobilized colistin gene *mcr-1* in 16% of the isolates, though 26% showed phenotypic resistance. Aside from the *mcr* gene, point mutations in the *pmrB* gene are also involved in phenotypic resistance to colistin. Other studies from Pakistan also showed the occurrence of the *mcr* gene in *E. coli* includes an 8% prevalence of *mcr-1* in *E. coli* isolated from Faisalabad poultry farms [56] and a 14% prevalence in APEC isolates from various regions of Pakistan [57]. Some previous studies from Pakistan have also reported the presence of *mcr-1* gene in clinical settings, healthy broilers, and migratory birds [58,59,60,61]. Moreover, a recent study from Bangladesh reported aa prevalence of 13.5% in *E. coli* samples isolated from various districts of Bangladesh [62] and a 36% prevalence of the *mcr-1* gene in the poultry sector of Eastern China [63].

The increase in the resistance against β-lactam antibiotics might be the result of the presence of β-lactam-inactivating enzymes called β-lactamases that are found in bacterial strains worldwide [64]. This study also proves this hypothesis, as more than 42.5% of *E. coli* isolates in this study harbored ESBL genes. Another study from Pakistan showed a 31% and 13.7% prevalence of ESBL-producing *E. coli* in cattle and chicken, respectively [65]. This situation is challenging, as these genes are plasmid-borne and can be transferred to other bacteria, environments, and humans [66].

It has been proposed that bacterial isolates with higher motilities and biofilm production are more resistant to antimicrobials and host defenses [67,68]. Biofilms help in acquiring new genetic material by horizontal gene transfer [69]. In this study, 104/200 isolates (52%) demonstrated some level of biofilm formation. Resistance to serum complement enhances the chances of extraintestinal infections by avoiding immune clearance [70]. In this study, thirty-six isolates (18%) were able to grow in human urine. This shows that these isolates had the potential to survive in high-urea and nitrogenous environments and might cause infection in animals and humans upon contact [24].

The prevalence of virulence genes was low, mainly because the isolates were isolated from the healthy animals. It is suggested that the *E. coli* isolates harboring *eae* and *stx* genes might represent a zoonotic risk, as these genes are often found in pathogenic *E. coli;* namely, enteropathogenic *E. coli* and enterohemorrhagic *E. coli* strains, respectively [48,71,72]. In this study, the *stx1* gene was present in our isolates (5%), whereas other DEC markers were not detected.

## 5. Conclusions

In conclusion, this study demonstrated the current state of antibiotic resistance in fecal *E. coli* isolates on the investigated dairy farms. A high prevalence of MDR (93%) isolates is alarming with penicillin, tetracycline, macrolide and fluoroquinolones being the least effective antibiotic classes. Although the prevalence of virulence associate genes was low, the isolates harboring the virulence genes were frequently MDR *E. coli*. These results propose that cows may represent an important reservoir of multidrug resistant *E. coli* and emphasize on the need of imposing the regulations for the proper use of antibiotics and growth promoters in food-producing animals.

## Figures and Tables

**Table 1:** Table representing the DEC pathotypes along with their detection marker.

Sr. No	DEC Pathotype	Detection Marker
1	Typical EPEC	*eaeA, bfpB*
2	Atypical EPEC	*eaeA*
3	EHEC	*stx1, stx2*
4	EAEC	*aggR*
5	EIEC	*invE*
6	ETEC	*It, stp, sth*

**Table 2:** Table presenting the percentage of enzymatic activities shown by bovine fecal *E. coli* isolates.

Enzymatic Activity	Confirmatory Test	
	No. of Isolates	Percentage %
ESBL	82	41
MBL	16	08
AmpC β-lactamase	06	03
Carbapenamase	02	01

## Data Availability

All data generated and analyzed during this study are included in this published article [and its Additional file].

## Supplementary Materials

The following supporting information can be downloaded at: https://www.mdpi.com/article/10.3390/antibiotics12010037/s1, Table S1: List of antibiotics used in this study along with their measure of each categorization; Table S2: List of primers used in this study. Refs. [33,39,73,74,75,76,77,78,79,80,81,82] are mentioned in the Supplementary Materials.
