# Supplementary material for: High Occurrence of Multidrug-Resistant Escherichia coli Strains in Bovine Fecal Samples from Healthy Cows Serves as Rich Reservoir for AMR Transmission
Source: Antibiotics (Basel). 2022 Dec 26;12(1):37. doi: 10.3390/antibiotics12010037 (PMC9855024; doi:10.3390/antibiotics12010037)
Supplement: Supplementary file 1 [file antibiotics-12-00037-s001.zip › antibiotics-2052809-supplementary.pdf]

Supplementary Table S1: List of antibiotics used in this study along with their measure of each categorization

S. No	Antibiotic	Abbreviation	Concentration (µg)	Sensitive (S)	Intermediate (I)	Resistant (R)
1.	Amoxicillin	AML	10	**≥ 17**	**14-16**	**≤ 13**
2.	Ampicillin	AMP	10	**≥ 17**	**14-16**	**≤ 13**
3 /.	Amoxicillin-clavulanate	AMC	20/10	**≥ 18**	**14-17**	**≤ 13**
4.	Doxycycline	DO	30	**≥ 14**	**11-13**	**≤ 10**
5.	Tetracycline	TE	30	**≥ 15**	**12-14**	**≤ 11**
6.	Piperacillin-tazobactam	TZP	100/10	**≥ 21**	**18-20**	**≤ 17**
7.	Eratapenem	ETP	10	**≥ 22**	**19-21**	**≤ 18**
8.	Meropenem	MEM	10	**≥ 23**	**20-22**	**≤ 19**
9.	Imipenem	IPM	10	**≥ 23**	**20-22**	**≤ 19**
10.	Gentamicin	CN	10	**≥ 15**	**13-14**	**≤ 12**
11.	Tobramycin	TOB	10	**≥ 15**	**13-14**	**≤ 12**
12.	Streptomycin	S	10	**≥ 15**	**12-14**	**≤ 11**
13.	Ciprofloxacin	CIP	05	**≥ 21**	**16-20**	**≤ 15**
14.	Norfloxacin	NOR	10	**≥ 17**	**13-16**	**≤ 12**
15.	Levofloxacin	LEV	05	**≥ 17**	**14-16**	**≤ 13**
16.	SXT	SXT	25	**≥ 16**	**11-15**	**≤ 10**
17.	Nitrofurantoin	F	300	**≥ 17**	**15-16**	**≤ 14**
18.	Erythromycin	E	15	**≥ 22**	**17-21**	**≤ 18**
19.	Ceftriaxone	CRO	30	**≥ 23**	**20-22**	**≤ 19**
20.	Cefotaxime	CTX	30	**≥ 26**	**23-25**	**≤ 22**
21.	Ceftazidime	CAZ	30	**≥ 21**	**18-20**	**≤ 17**
22.	Amikacin	AK	30	**≥ 17**	**15-16**	**≤ 14**
23.	Cefepime	FEP	30	**≥ 25**	**19-24**	**≤ 18**
24.	Cefoxitin	FOX	30	**≥ 18**	**15-17**	**≤ 14**
25.	Chloramphenicol	C	30	**≥ 18**	**13-17**	**≤ 12**
26.	Ofloxacin	OFX	05	**≥ 16**	**13-15**	**≤ 12**

Supplementary Table S2: List of primers used in this study

Sr. No	Gene		Primers (5’-3’)	Amplicon Size (bp)	Nucleotides	Reference
1.	***uidA***	F	CCCTTACGCTGAAGAGATGC	401	20	This Study
		R	GGCACAGCACATCAAAGAGA		20	
2.	***chuA***	F	GACGAACCAACGGTCAGGAT	279	20	01
		R	TGCCGCCAGTACCAAAGACA		20	
3.	***yjaA***	F	TGAAGTGTCAGGAGACGCTG	211	20	01
		R	ATGGAGAATGCGTTCCTCAAC		21	
4.	**TSPE4.C2**	F	GAGTAATGTCGGGGCATTCA	152	20	01
		R	CGCGCCAACAAAGTATTACG		20	
5.	***mcr-1***	F	AGTCCGTTTGTTCTTGTGGC	320	20	02
		R	AGATCCTTGGTCTCGGCTTG		20	
6.	***mcr-2***	F	CAAGTGTGTTGGTCGCAGTT	715	20	02
		R	TCTAGCCCGACAAGCATACC		20	
7.	***blaKPC***	F	TGTCACTGTATCGCCGTC	900	18	03
		R	CTCAGTGCTCTACAGAAAACC		21	
8.	***blaIMP***	F	GAAGGCGTTTATGTTCATAC	587	20	04
		R	GTACGTTTCAAGAGTGATGC		20	
9.	***blaVIM***	F	GTTTGGTCGCATATCGCAAC	389	20	04
		R	AATGCGCAGCACCAGGATAG		20	
10.	***blaNDM***	F	GCAGCTTGTCGGCCATGCGGGC	782	22	05
		R	GGTCGCGAAGCTGAGCACCGCAT		23	
11.	***blaOXA-48-like***	F	GCGTGGTTAAGGATGAACAC	438	20	06
		R	CATCAAGTTCAACCCAACCG		20	
12.	***blaSHV***	F	ATGCGTTATATTCGCCTGTG	747	20	07
		R	TGCTTTGTTATTCGGGCCAA		20	
13.	***blaTEM***	F	TCGCCGCATACACTATTCTCAGAATGA	445	27	07
		R	ACGCTCACCGGCTCCAGATTTAT		23	
14.	***blaCTX***	F	ATGTGCAGYACCAGTAARGTKATGGC	593	26	07
		R	TGGGTRAARTARGTSACCAGAAYCAGCGG		29	

15.	***iss***	F	GTTATTTTCTGCCGCTCTGG	**227**	20	This Study
		R	AACCGAGCAATCCATTTACG		20	
16.	***papC***	F	AATAAAAACGTGGCGGACTG	440	20	This Study
		R	TATCCTTTCTGCAGGGATGC		20	
17.	***cvaC***	F	CCTCCTACCCTTCACTCTTG	501	20	This Study
		R	GGATGGAGACATTGCAGGAT		20	
18.	***kpsMT III***	F	TCCTCTTGCTACTATTCCCCCT	392	22	08
		R	AGGCGTATCCATCCCTCCTAAC		22	
19.	***papA***	F	ATGGCAGTGGTGTTTTGGTG	717	20	08
		R	CGTCCCACCATACGTGCTCTTC		22	
20.	***fimH***	F	TCGAGAACGGATAAGCCGTGG	508	21	08
		R	GCAGTCACCTGCCCTCCGGTA		21	
21.	***papEF***	F	GCAACAGCAACGCTGGTTGCATCAT	326	25	08
		R	AGAGAGAGCCACTCTTATACGGACA		25	
22.	***ireA***	F	GATGACTCAGCCACGGGTAA	254	20	08
		R	CCAGGACTCACCTCACGAAT		20	
23.	***ibeA***	F	AGGCAGGTGTGCGCCGCGTAC	171	21	08
		R	TGGTGCTCCGGCAAACCATGC		21	
24.	***PAI***	F	GGACATCCTGTTACAGCGCGCA	925	22	08
		R	TCGCCACCAATCACAGCCGAAC		22	
25	***cnf-I***	F	ATCTTATACTGGATGGGATCATCTTGG	1105	27	08
		R	GCAGAACGACGTTCTTCATAAGTATC		26	
26.	***fyuA***	F	TGATTAACCCCGCGACGGGAA	787	21	08
		R	CGCAGTAGGCACGATGTTGTA		21	
27.	***iroN***	F	AAGTCAAAGCAGGGGTTGCCCG	667	22	08
		R	GATCGCCGACATTAAGACGCAG		22	
28.	***bmaE***	F	ATGGCGCTAACTTGCCATGCTG	507	22	08
		R	AGGGGGACATATAGCCCCCTTC		22	
29.	***sfa***	F	CTCCGGAGAACTGGGTGCATCTTAC	410	25	08
		R	CGGAGGAGTAATTACAAACCTGGCA		25	
30.	***iutA***	F	GGCTGGACATCATGGGAACTGG	302	22	08

		R	CGTCGGGAACGGGTAGAATCG		21	
31.	***papG***	F	GGCCTGCAATGGATTTACCTGG	258	22	08
		R	CCACCAAATGACCATGCCAGAC		22	
32.	***kpsMT* (K1)**	F	TAGCAAACGTTCTATTGGTGC	153	21	08
		R	Used with *kpsM II* Reverse		23	
33.	***foc***	F	CAGCACAGGCAGTGGATACGA	364	21	08
		R	GAATGTCGCCTGCCCATTGCT		21	
34.	***afa***	F	GGCAGAGGGCCGGCAACAGGC	594	21	08
		R	CCCGTAACGCGCCAGCATCTC		21	
35.	***kpsM II***	F	GCGCATTTGCTGATACTGTTG	272	21	08
		R	CATCCAGACGATAAGCATGAGCA		23	
36.	***invE***	F	CGATAGATGGCGAGAAATTATATCCCG	766	27	09
		R	CGATCAAGAATCCCTAACAGAAGAATCAC		29	
37.	***aggR***	F	ACGCAGAGTTGCCTGATAAAG	400	21	09
		R	AATACAGAATCGTCAGCATCAGC		23	
38.	***bfpB***	F	GACACCTCATTGCTGAAGTCG	910	21	09
		R	CCAGAACACCTCCGTTATGC		21	
39.	***lt***	F	GAACAGGAGGTTTCTGCGTTAGGTG	655	25	09
		R	CTTTCAATGGCTTTTTTTTGGGAGTC		26	
40.	***stp***	F	CCTCTTTTAGYCAGACARCTGAATCASTTG	157	30	09
		R	CAGGCAGGATTACAACAAAGTTCACAG		27	
41.	***sth***	F	TGTCTTTTTCACCTTTCGCTC	171	21	09
		R	CGGTACAAGCAGGATTACAACAC		23	
42.	***stx1***	F	ACACTGGATGATCTCAGTGG	614	20	10
		R	CTGAATCCCCCTCCATTATG		20	
43.	***stx2***	F	CCATGACAACGGACAGCAGTT	779	21	10
		R	CCTGTCAACTGAGCAGCACTTTG		23	
44.	***eaeA***	F	GTGGCGAATACTGGCGAGACT	890	21	11
		R	CCCCATTCTTTTTCACCGTCG		21	
45.	***hlyA***	F	ACGATGTGGTTTATTCTGGA	165	20	12

		R	CTTCACGTGACCATACATAT		20	

1. Clermont, O., Stéphane, B. & Edouard, B. Rapid and simple determination of the *Escherichia coli* phylogenetic group. *Appl. Environ. Microbiol.* **66**(10), 4555–4558, https://doi.org/10.1128/AEM.66.10.4555-4558.2000 (2000).
2. Rebelo, A.R., Bortolaia, V., Kjeldgaard, J.S., Pedersen, S.K., Leekitcharoenphon, P., Hansen, I.M., Guerra, B., Malorny, B., Borowiak, M., Hammerl, J.A., Battisti, A., Franco, A., Alba, P., Perrin-Guyomard, A., Granier, S. A., De Frutos Escobar, C., Malhotra-Kumar, S., Villa, L., Carattoli, A., & Hendriksen, R.S. Multiplex PCR for detection of plasmid-mediated colistin resistance determinants, *mcr-1, mcr-2, mcr-3, mcr-4* and *mcr-5* for surveillance purposes. *Euro surveillance : bulletin Europeen sur les maladies transmissibles = European communicable disease bulletin*, **23**(6), 17-00672, https://doi.org/10.2807/1560-7917.ES.2018.23.6.17-00672 (2018).
3. Yigit, H., Queenan, A.M., Anderson, G.J., Domenech-Sanchez, A., Biddle, J.W., Steward, C.D., Alberti, S., Bush, K., & Tenover, F.C. Novel carbapenem-hydrolyzing beta-lactamase, *KPC-1*, from a carbapenem-resistant strain of *Klebsiella pneumoniae*. *Antimicrob. Agents Chemother.* **45**(4), 1151–1161, https://doi.org/10.1128/AAC.45.4.1151-1161.2001 (2001).
4. Pitout, J. D., Gregson, D.B., Poirel, L., McClure, J.A., Le, P. & Church, D.L. Detection of *Pseudomonas aeruginosa* producing metallo-beta-lactamases in a large centralized laboratory. *J. Clin. Microbiol.* **43**(7), 3129–3135, https://doi.org/10.1128/JCM.43.7.3129-3135.2005 (2005).
5. Peirano, G., Ahmed-Bentley, J., Woodford, N. & Pitout, J. D. New Delhi metallo-beta-lactamase from traveler returning to Canada. *Emerg. Infect. Dis*. **17**(2), 242–244, https://doi.org/10.3201/eid1702.101313 (2011).
6. Poirel, L., Walsh, T.R., Cuvillier, V. & Nordmann, P. Multiplex PCR for detection of acquired carbapenemase genes. *Diagn. Microbio.l Infect. Dis*. **70**(1), 119-23. doi: 10.1016/j.diagmicrobio.2010.12.002 (2011).
7. Monstein, H.J., Ostholm-Balkhed, A., Nilsson, M.V., Nilsson, M., Dornbusch, K., Nilsson, L.E. Multiplex PCR amplification assay for the detection of ^{bla}*SHV*, ^{bla}*TEM* and ^{bla}*CTX-M* genes in Enterobacteriaceae. APMIS **115**(12), 1400-1408, doi: 10.1111/j.1600-0463.2007.00722.x. (2007).
8. Johnson J.R. & Stell A.L. Extended virulence genotypes of *Escherichia coli* strains from patients with urosepsis in relation to phylogeny and host compromise. *J. Infect. Dis.* **181**,261–272, (2000).
9. Yun, Z., Zeng, L., Huang, W. *et al.* Detection and Categorization of Diarrheagenic *Escherichia coli* with Auto-microfluidic Thin-film Chip Method. *Sci Rep* **8,** 12926, https://doi.org/10.1038/s41598-018-30765-3 (2018).

10. Gannon, V.P.J., King, R.K., Kim, J.Y. & Golsteyn Thomas, E.J. Rapid and sensitive method for detection of Shiga-like toxin-producing *Escherichia coli* in ground beef using the polymerase chain reaction. *Appl. Environ. Microbiol.* **58**, 3809–3815, (1992).
11. Gannon, V.P.J, Souza, S.D., Graham, T., King, R.K., Rahn, K. & Read, S. Use of the flagellar H7 gene as a target in multiplex PCR assays and improved specificity in identification of enterohemorrhagic *Escherichia coli* strains. *J Clin Microbiol.* **35**, 656–662, (1997).
12. Fratamico, P.M., Sackitey, S.K., Wiedmann, M. & Deng, M.Y. Detection of *Escherichia coli* O157:H7 by multiplex PCR. *J Clin Microbiol.* **33**, 2188–2191, (1995).
